# Utility of forensic detection of rabies virus in decomposed exhumed dog carcasses

**DOI:** 10.4102/jsava.v86i1.1220

**Published:** 2015-05-18

**Authors:** Wanda Markotter, Jessica Coertse, Kevin le Roux, Joey Peens, Jacqueline Weyer, Lucille Blumberg, Louis H. Nel

**Affiliations:** 1 Department of Microbiology and Plant Pathology, University of Pretoria, South Africa; 2 Allerton Provincial Veterinary Laboratory, Pietermaritzburg, South Africa; 3 State Veterinary Office, Department of Agriculture and Environmental Affairs, South Africa; 4 Centre for Emerging and Zoonotic Diseases, National Institute for Communicable Diseases of the National Health Laboratory Services, South Africa

## Abstract

This report describes four suspected rabies cases in domestic dogs that were involved in human exposures. In all these cases, the animals were buried for substantial times before rabies testing was performed. Animal rabies is endemic in South Africa and domestic dogs are the main vector for transmission to humans. Diagnosis of rabies in humans is complicated, and diagnosis in the animal vector can provide circumstantial evidence to support clinical diagnosis of rabies in humans. The gold standard diagnostic method, fluorescent antibody test (FAT), only delivers reliable results when performed on fresh brain material and therefore decomposed samples are rarely submitted for diagnostic testing. Severely decomposed brain material was tested for the presence of rabies virus genomic material using a quantitative real-time reverse transcription polymerase chain reaction (q-real-time RT-PCR) assay when conventional molecular methods were unsuccessful. This may be a useful tool in the investigation of cases where the opportunity to sample the suspected animals *post mortem* was forfeited and which would not be possible with conventional testing methodologies because of the decomposition of the material.

## Introduction

Rabies is a fatal and zoonotic disease associated with up to 55 000 human cases per annum worldwide. Many of these cases occur in African countries, where rabies remains uncontrolled in domestic dogs (Knobel *et al*. 2005). The disease is likely underestimated in African countries because of misdiagnosis and lack of capacity to investigate cases by laboratory testing (Blumberg *et al*. 2007). Rabies remains endemic in South Africa, where up to 30 human cases are reported annually (Weyer *et al*. 2011). The disease also continues to re-emerge in localities where it was previously well controlled, including Limpopo, Gauteng and Mpumalanga provinces (Mkhize *et* *al*. 2010; Sabeta *et al*. 2013; Sabeta, Mkhize & Ngoepe 2011).

The majority of animal cases diagnosed occur in the hyperendemic KwaZulu-Natal (KZN) province and neighbouring Mpumalanga and Eastern Cape provinces, where the main vectors are domestic dogs. Rabies control efforts in KZN have been boosted in recent years with support through a Bill and Melinda Gates/World Health Organization (WHO) initiative (Nel 2013). Subsequently, no human cases were diagnosed from June 2010 to September 2011 – which constituted the first time in more than 20 years that KZN did not report a human rabies case for a period longer than one year. The gold standard for rabies diagnosis remains the fluorescent antibody test (FAT) (Dean, Abelseth & Atansiu 1996). As brain material is required for this test, diagnosis can only be performed *post mortem* and the FAT yields the most reliable results when performed on fresh brain material. When decomposed brain material is used, the sensitivity of the test is reduced and may even result in false negatives (Robardet *et al*. 2011). In severely decomposed material there is also no intact brain available and samples are received as a liquid substance that is unsuitable for FAT testing. In some cases, conventional reverse transcription polymerase chain reaction (RT-PCR) and heminested RT-PCR (hnRT-PCR) assays have proven to be useful when other methods such as FAT were not successful (David *et al*. 2002; Kamolvarin *et al*. 1993; McElhinney *et al*. 2014). However, these methods target a comparatively large (over 350 base pair) region of the nucleoprotein gene of the viral genome and in cases of advanced decomposition the viral ribonucleic acid (RNA) may have deteriorated to the extent that these methods are no longer successful. Real-time RT-PCR, in contrast to conventional molecular methods, targets a shorter conserved region of the nucleoprotein gene of the viral genome. Furthermore, quantitative real-time RT-PCR (q-real-time RT-PCR) has previously been shown to be successful for detection of rabies viral RNA in formalin-fixed brain material when other methods have been unsuccessful (Coertse *et al*. 2011). In 2012, two human cases of rabies from KZN were investigated by the National Institute for Communicable Diseases of the National Health Laboratory Services (NICD-NHLS), South Africa. In both cases, *ante-mortem* testing for rabies proved problematic because of the rabies vaccination histories of the patients, although incomplete prophylaxis was provided (Mollentze *et* *al*. 2013). The dogs associated with the possible exposures died after the exposure events but were buried, and no specimens were submitted for laboratory investigation. To provide circumstantial evidence to support the clinical diagnosis of rabies in these patients, the implicated dogs were exhumed and submitted for laboratory investigation.

In this report, three cases are described in which a q-real-time RT-PCR assay was applied for rabies diagnosis in exhumed dogs when other methods were unsuccessful because of the state of decomposition of the brain material.

## Case presentation

### Case history

#### Case 1

On 2 May 2012, a 29-year-old male farmer from Underberg was admitted to a hospital in Pietermaritzburg, KZN. The patient reported contact with a stray puppy some two months before the onset of symptoms. The patient provided shelter for the puppy, but after a few days the animal developed symptoms which in retrospect could have been considered consistent with rabies. The dog subsequently died and was buried on the farm. After consideration of the patient history, rabies was deemed likely. Saliva, skin and cerebrospinal fluid (CSF) were collected over the course of his illness and sent to the NICD-NHLS in Johannesburg but were consistently negative for the presence of rabies virus RNA using conventional as well as real-time RT-PCR methods. To further investigate, the puppy (referred to as Dog GA) was exhumed and the decomposed brain material sent to the University of Pretoria in 50% glycerol-saline solution for molecular testing on 11 May 2012. Rabies-specific IgG was detected in the serum of the patient; however, this was likely as a result of the vaccine he received upon admission. Initial rabies-specific serological tests on CSF were negative. Subsequent CSF samples collected over four weeks indicated the presence of rabies-specific IgG at low titres. The FAT confirmed the presence of rabies virus antigen in a *post-mortem* brain biopsy specimen of the patient. Real-time RT-PCR was also performed on this specimen at the NICD-NHLS and the product sequenced (referred to as SPU 134/12).

#### Case 2

On 20 May 2012, a 4-year-old child was admitted to a local hospital in KZN. The admitting doctor noticed symptoms suggestive of rabies virus infection, including general weakness, loss of appetite and confusion. Upon retrospective investigation it was found that the child was scratched and bitten by two different dogs on two separate occasions. The first incident took place on 29 March 2012, when a neighbour’s dog (referred to as Dog MK) scratched the child on the forehead whilst the child was playing outside. The parents of the child did not seek medical attention. On the same day, Dog MK bit a 5-month-old puppy (referred to as Dog ZC) belonging to another neighbour. In the week after the scratch incident, the owners of Dog MK noticed the dog behaving strangely, including excessive salivation and barking, loose jaw and a weak gait. Dog MK died on 05 April 2012 and was buried by the owner. Dog ZC, the puppy bitten by Dog MK, attacked and bit the 4-year-old child on the right ankle on 23 April 2012 whilst the child was walking. Dog ZC was killed and buried by the owner on the same day of the biting incident. On 28 May 2012, three saliva samples and a nuchal biopsy specimen were collected from the patient and sent to the NICD-NHLS but were negative for the presence of viral RNA. It was decided to exhume both dogs involved in this case for rabies testing. The brain material was subsequently sent to the University of Pretoria in 50% glycerol-saline solution for molecular testing on 20 June 2012.

#### Case 3

On 28 August 2012, a 21-year-old male was admitted to a local hospital in KZN. Upon investigation, it was found that a dog (referred to as Dog VG) bit the patient on 19 July 2012 whilst he was visiting his girlfriend in the Tshelimnyama area. The owners of Dog VG mentioned that the dog was usually well behaved but suddenly started showing strange behaviour and that the dog was not vaccinated during the recent vaccine campaign in the area. Following these events, the owner chained the dog; however, the dog broke free and went missing. Other people from the neighbourhood reported seeing Dog VG attacking other dogs on several occasions. Dog VG was killed during one of these attacks. The carcass of Dog VG was found slightly submerged in water and in an advanced state of decomposition on 28 August 2012. Brain material was subsequently sent to the University of Pretoria in 50% glycerol-saline solution for molecular testing on 30 August 2012.

### Molecular diagnosis

**FIGURE 1:**
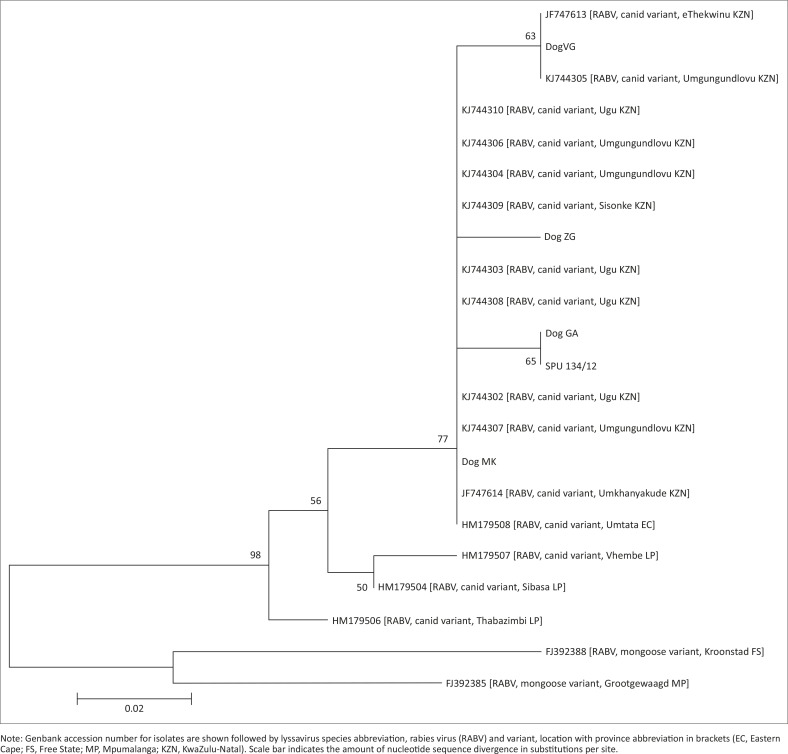
Neighbour-joining phylogenetic tree constructed from a 70 bp sequence (position: 564-632) of the nucleoprotein gene (numbered according to the Pasteur virus sequence, Genbank accession no. M13215) of isolates obtained from decomposed dog brain material (Dog GA, Dog MK, Dog ZC, Dog VG), the human *post-mortem* brain material (SPU134/12) and other rabies sequences (Table 1).

The dogs in these cases were buried for 30–76 days before being exhumed for rabies testing. The FAT was not attempted because of the severe decomposition of the samples, with no intact brain material present in the samples to prepare a smear for FAT. Any results may be unreliable because of the decrease in sensitivity of the FAT when using decomposed material, so it was decided to proceed with molecular testing only. Total RNA was extracted from a decomposed sample from the exhumed dogs by using Trizol reagent (Invitrogen, USA), according to the manufacturer’s instructions. Conventional RT-PCR and hnRT-PCR were performed as previously described (Coertse *et al*. 2010) and were consistently negative. Quantitative real-time RT-PCR (Coertse *et al*. 2010) was performed on 1 µL RNA and yielded positive results in all four cases. Average viral RNA copy numbers were as follows: Dog GA 50 copies/reaction, Dog MK 213 copies/reaction, Dog ZC 733 copies/reaction and Dog VG 6.5 × 10^6^ copies/reaction. The amplicons were purified and sequenced and phylogenetic analysis was performed (Markotter *et al*. 2006) with representative rabies virus sequences available in the public domain (Table 1). Although the resolution after phylogenetic analysis was not ideal as a result of the short region of sequence available from the real-time RT-PCR amplicon, the resulting neighbour-joining (NJ) phylogenetic analysis (Figure 1) confirmed the expected relationship with rabies virus circulating in the KZN domain. The patient bitten by Dog GA died and a viral sequence was generated from samples obtained *post mortem*. This viral sequence (SPU134/12) was identical to the viral sequence obtained from Dog GA (Figure 1).

**TABLE 1:** Representative rabies viruses from South Africa included in the phylogenetic analysis.

Genbank accession number	Virus	Host	Location	Year
KJ744307	RABV, canid variant	Domestic dog (*Canis familiaris*)	Umgungundlovu, KwaZulu-Natal	2011
KJ744310	RABV, canid variant	Domestic dog (*Canis familiaris*)	Ugu, KwaZulu-Natal	2011
KJ744306	RABV, canid variant	Domestic dog (*Canis familiaris*)	Umgungundlovu, KwaZulu-Natal	2011
KJ744305	RABV, canid variant	Domestic dog (*Canis familiaris*)	Umgungundlovu, KwaZulu-Natal	2011
KJ744304	RABV, canid variant	Domestic dog (*Canis familiaris*)	Umgungundlovu, KwaZulu-Natal	2010
KJ744309	RABV, canid variant	Caprine	Sisonke, KwaZulu-Natal	2010
KJ744303	RABV, canid variant	Domestic dog (*Canis familiaris*)	Ugu, KwaZulu-Natal	2010
KJ744308	RABV, canid variant	Domestic dog (*Canis familiaris*)	Ugu, KwaZulu-Natal	2010
KJ744302	RABV, canid variant	Domestic dog (*Canis familiaris*)	Ugu, KwaZulu-Natal	2010
JF747613	RABV, canid variant	Domestic dog (*Canis familiaris*)	eThekwini, KwaZulu-Natal	2008
JF747614	RABV, canid variant	Domestic dog (*Canis familiaris*)	Umkhanyakude, KwaZulu-Natal	2008
HM179504	RABV, canid variant	Domestic dog (*Canis familiaris*)	Sibasa, Limpopo	2006
HM179508	RABV, canid variant	Domestic dog (*Canis familiaris*)	Umtata, Eastern Cape	2005
HM179507	RABV, canid variant	Black-backed jackal (*Canis mesomelas*)	Vhembe, Limpopo	2005
HM179506	RABV, canid variant	Domestic dog (*Canis familiaris*)	Thabazimbi, Limpopo	1996
FJ392388	RABV, mongoose variant	Mongoose (*Cynictis penicillata*)	Kroonstad, Free State	1995
FJ392385	RABV, mongoose variant	Mongoose (*Cynictis penicillata*)	Grootgewaagd, Mpumalanga	1990

RABV, Rabies virus.

## Discussion

Three possible human exposures to rabies virus after contact with domestic dogs are described. In all these cases, the dogs had already been buried for a substantial time (up to 76 days) when rabies in the humans was first suspected. In none of the human cases could rabies be confirmed *ante mortem*, which complicated patient care. The dogs were exhumed and brain material tested using a real-time RT-PCR method. The utility of the real-time RT-PCR protocol in the diagnosis and molecular characterisation of rabies virus RNA from exhumed dog brain material was demonstrated where other methods were unsuccessful. This indicates the importance of using a sensitive real-time PCR assay in suspected rabies cases where other methods are unreliable or will fail because of decomposition of the sample and degradation of the viral RNA. Deoxyribonucleic acid (DNA) sequencing could also be performed on this small amplicon and phylogenetic analysis was used to indicate the epidemiological relationships of these viruses. Conclusive diagnosis in the animal vectors in each of these cases was helpful in eliminating the differential diagnoses in the patients and in providing the appropriate patient care. In addition, the positive diagnosis in dogs was important with regard to re-evaluation of the dog rabies control measures in the affected areas.

## Conclusion

A real-time RT-PCR assay was applied in cases where conventional methods were unsuccessful. This method was shown to be very effective for *post-mortem* rabies diagnosis in severely decomposed carcasses. Subsequent phylogenetic analysis also provided epidemiological information that might otherwise not have been known.
